# Editorial: Computational Methods in Inferring Cancer Tissue-of-Origin and Cancer Molecular Classification

**DOI:** 10.3389/fgene.2021.644542

**Published:** 2021-02-23

**Authors:** Ling Kui, Cheng Guo, Shuai Cheng Li, Jialiang Yang, Min Tang

**Affiliations:** ^1^School of Pharmacy, Jiangsu University, Zhenjiang, China; ^2^Harvard Medical School, Dana-Farber Cancer Institute, Boston, MA, United States; ^3^Center for Infection and Immunity, School of Public Health, Columbia University, New York, NY, United States; ^4^Department of Computer Science, City University of Hong Kong, Kowloon, China; ^5^Geneis (Beijing) Co. Ltd., Beijing, China; ^6^School of Life Sciences, Jiangsu University, Zhenjiang, China

**Keywords:** cancer tissue-of-origin, cancer molecular classification, liquid biopsy, machine learning, single-cell

The development of cancer therapeutics increasingly relies on the results of tissue-of-origin and molecular classification. In the clinic, up to 5% of the cancer primary site is unclassified (CUP). For clinicians, it is important to identify the sensitive patients and determine treatment. The main option is empirical chemotherapy, which leads to a lower survival rate. Therefore, inferring cancer tissue-of-origin is an urgent need to be solved. The key point is to detect the exact genetic events associated with cancer formation, which usually contribute to cell proliferation and uncontrolled metabolic changes. However, using only experimental approaches cannot provide a full view of the genetic features in the era of big biomedical data. Although a series of computational methods have been developed in this area, the accuracy is often insufficient for clinical use.

The molecular classification in cancer is useful in optimizing treatment policies. With data accumulation, especially more and more single-cell sequencing data, the molecular classification will be improved for various cancer types. As better biomarkers evolve, more efficient treatments and new drugs will be developed.

This Research Topic gathered research articles and reviews representing not only the computational methods for inferring the origins and molecular classification but also translational studies for cancer treatment in hospitals. This collection of papers sheds light on the development of cancer therapeutics, with a focus on the most cutting-edge computational applications in cancer diagnosis.

The 19 published articles consist of 18 research papers and a regular review, which comprehensively illustrates the use of computational methods in inferring cancer Tissue-of-Origin and molecular classification in various cancer types, including but not limited to hepatocellular carcinoma (HCC), Pancreatic cancer (PC), ovarian cancer (OC), glioma, gastric cancer (GC), circulating tumor cells (CTCs), cervical cancer (CC), and endometrial cancer (EC).

Seven research articles introduce several different methods to capture gene signature (models) for similar purposes. Li et al. first employed the limma R package to the got the top 5,000 significant differentially expressed genes (DGEs) in HC. These DEGs were gathered into nine modules after they underwent a weighted correlation network analysis (WGCNA). Then, six genes were screened by univariate, LASSO, and multivariate Cox regression analysis, and they were validated as an independent prognostic factor in survival analysis (Li et al.). Most of the bioinformatic approaches in this study were implemented in the article of Zhang et al., whose aim was to develop a stemness index-based gene signature for lower-grade glioma (LGG). Interestingly, the same research group developed an immune-related signature for prognosis prediction and risk stratification in LGG with data from The Cancer Genome Atlas (TCGA), Genome Tissue Expression (GTEx), and Chinese Glioma Genome Atlas (CGGA) (Zhang et al.). A similar study in CC and EC was completed by Ding et al. Importantly, they validated the gene signature with many methods, such as enrichment analyses through GO, KEGG, and GSEA pathways, Kaplan-Meier survival curve, ROC curves, and immune cell infiltration (Ding et al.). Moreover, Pan et al. also demonstrated that gene methylation can be utilized to classify gliomas as signatures. They used advanced computational methods of Monte Carlo feature selection (MCFS), incremental feature selection (IFS), and support machine vector (SVM) to detect methylation features related to glioma subclasses (Pan et al.). A back-to-back study performed by Hou et al. illustrated the functions and mechanisms of N6-methyladenosine (m6A) modification in the development of PC. A six-m6A-regulator-signature related to overall survival (OS) was identified by LASSO regression (Hou et al.). Furthermore, Kieffer et al. established gene signatures by combining transcriptomic and genomic data for high-grade OC.

Notably, three research articles elucidate the application of machine learning in gene feature captivation. Using DNA somatic mutation data, Liu et al. extracted genetic features using the random forest algorithm and established a logistic regression-based classifier. With the extracted matrix of features from the functional 300 genes, the prediction accuracy can reach up to 81% in 10-fold cross-validation. To reduce the workload of CTCs counting and improve the automation level, He et al. established a cell recognition program based on deep learning to identify the CTCs. In their project, the CTCs images of 600 in-house patients were analyzed with python's OpenCV scheme for segmentation. Then, convolutional neural network deep learning networks in machine learning algorithms were implemented on 1,300 cells for training, and the others were used for testing. The final specificity and sensitivity of recognition reached 91.3 and 90.3%, respectively (He et al.). Qian et al. provide a feature extraction algorithm based on Support Vector Machines (SVM) for cancer lectins prediction with a fusion of G-Gap dipeptide.

Three research articles focus on the development of computational approaches. Zhu et al. exploited a prediction model called MiRNA-Disease Association prediction (BHCMDA) based on the Biased Heat Conduction (BHC) algorithm to discover potentially associated miRNAs of diseases by integrating known miRNA-disease associations, the disease semantic similarity, the miRNA functional similarity, and the Gaussian interaction profile kernel similarity. Zhao et al. created a novel computational approach named multiplex biological network (MON) by integrating protein interaction networks (PINs), protein domains, and gene expression files. The new approach was able to detect the essential proteins by extending the random walk with a restart algorithm to the tensor (Zhao et al.). To predict lung cancer recurrence after surgical resection, Wu et al. established a convolutional neural network (CNN) framework called DeepLRHE by analyzing histopathological images of patients from the TCGA database, and the receiver operating characteristic (ROC) curve (AUC) was 0.79.

Finally, the systematic review demonstrates in detail that the CLDN18-ARHGAP fusion is a significant molecular characteristic of diffuse GC, which is also an independent prognostic risk factor (Zhang et al.).

All of the research articles and reviews in this Research Topic use state-of-the-art sources about the origin and gene signatures of different cancers, examining the available computational methods and providing a guide for physicians.

## Author Contributions

This editorial was designed by MT and written by LK and CG. SL and JY revised it. All authors made a direct and intellectual contribution to this topic and approved the article for publication.

## Conflict of Interest

JY was employed by the company Geneis Co. Ltd. (Beijing). The remaining authors declare that the research was conducted in the absence of any commercial or financial relationships that could be construed as a potential conflict of interest.

